# Culture independent DNA extraction method for bacterial cells concentrated from water

**DOI:** 10.1016/j.mex.2022.101653

**Published:** 2022-03-02

**Authors:** K.B. Hoorzook, T.G. Barnard

**Affiliations:** University of Johannesburg, South Africa

**Keywords:** Celite, Environmental water, *Escherichia coli*, Genomic and plasmid extraction, Guanidium thiocyanate, Membrane filtration

## Abstract

• To bypass the problem of viable but non-culturable bacteria that cannot be isolated by culturable methods would be to isolate DNA from bacterial cells concentrated from water samples used as a template for the polymerase chain reactions (PCR). DNA extraction protocol (Omar et al. 2010) was used as a foundation for extracting *Escherichia coli* DNA from water. The method combinations i.e., guanidium thiocyanate, celite and home-made spin column were chosen because it has been shown to be reliable, rapid, simple, and inexpensive for routine analysis in developing country settings.
• The following optimizations were included: (a) Polycarbonate (Poly) was statistically compared with Polyether sulphone (PES), Nitrocellulose acetate (NA) and Nitrocellulose (NC) membranes; (b) Various housing containers for the membranes were tested: plastic/glass petri-dish, Falcon tubes, Ogreiner cryovials; (c) various solutions was tested to add to the membrane to remove cells from membranes; (d) celite was chosen to bind the DNA because it had a higher DNA binding capacity compared to silicon dioxide; (e) incubation times and rotation speed were tested when adding reagents.
• The optimized in-house DNA extraction method was validated with environmental water samples, high (dam water) and low (borehole) bacterial load to determine upper and lower detection limits of the method.

## Graphical abstract

**Figure:**
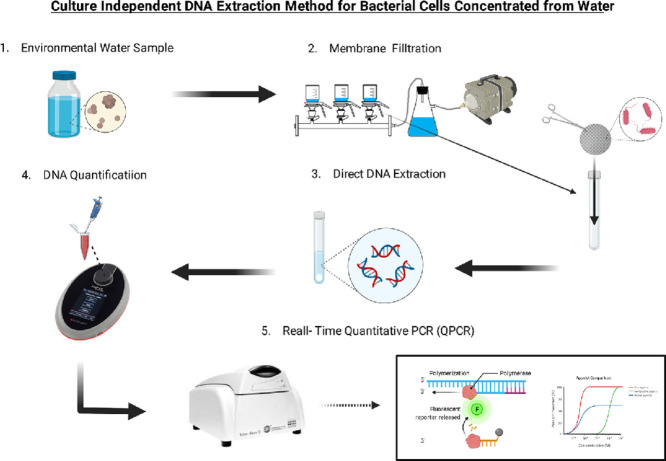

Specifications table

**Table:** 

Subject area;	Biochemistry, Genetics and Molecular Biology
More specific subject area;	DNA extraction
Method name;	Culture independent DNA extraction method
Name and reference of original method;	(1) Boom et al. (1990) Rapid and Simple Method for Purification of Nucleic Acids. Journal Of Clinical Microbiology, 28: 495-503. (2) Borodina et al. (2003) DNA purification on homemade silica spin columns, Journal of Analytical Biochemistry, 321: 135-137. (3) Omar et al. (2010) Development of a competitive PCR assay for the quantification of total *Escherichia coli* DNA in water, African Journal Of Biotechnology, 9 (4): 564-572.
Resource availability;	Logbooks, doctorate dissertation, UJ repository

M**ethodology**

## Growth and maintenance of bacterial strains

A commensal non-pathogenic *E. coli* (ComEC) and pathogenic Entero-haemorrhagic *E. coli* (EHEC) strain (ESCCO 21) was used in this study. These strains were cultured from frozen glycerol stocks on Plate Count Agar (PCA) (Oxoid, UK) and incubated under aerobic conditions at 37 °C for 16 h. Single colonies were enriched in 100 mℓ nutrient broth and incubated under aerobic conditions at 37 °C for 16 h with rotation at 200 rpm.

## Buffer composition and preparation

The preparation of the celite, Lysis buffer L6, Lysis buffer L7a, Washing buffer and spin columns used for the “Optimised in-house DNA extraction method” is as follows:

### Celite

Celite (Sigma Aldrich) was prepared by suspending 10 g in 50 mℓ distilled water and adding 500 μℓ hydrochloric acid (HCl) (32% w/v) to the solution. Thereafter it was sterilised for 15 min at 121 °C and the bottle wrapped in aluminium foil (celite solution is sensitive to light) and refrigerated at -20 °C (stable for 3 weeks at room temperature) [1].

### Lysis buffer (L6)

Lysis buffer L6 was prepared by dissolving 120 g guanidinium thiocyanate (GuSCN) (G6639) in 100 mℓ of 0.1 M hydroxymethyl amino methane-hydrochloric acid (Tris-HCl) (pH 6.4) in a 500 mℓ Schott bottle. The bottle was heated to 60°C to dissolve the GuSCN. If not heated the GuSCN will not dissolve. After heating, 22 mℓ of a 0.2 Methylenediamine tetra-acetate (EDTA) (pH 8.0) with 2.6 mℓ triton X-100 (Sigma Aldrich) solution was added to the suspension. The suspension was mixed and dispensed into 50 mℓ Eppendorf tubes and 0.5 mℓ of celite suspension (Section 2.1) was added to remove any contaminating DNA from the buffer. The final solution was left to stand for at least 1 h at room temperature with sporadic mixing. The celite was pelleted by centrifugation at 3000 rpm for 10 min (NeoFuge-15R, Heal Force, Vacutec®) and the supernatant was transferred into sterile 50 mℓ Eppendorf tubes wrapped in aluminium foil (sensitivity towards light) (stable 3 weeks at room temperature).

### Lysis buffer (L7A)

Buffer L7A was prepared from buffer L6 by the addition of α-casein (Sigma Aldrich) to a final concentration of 1 mg/mℓ from a 50X (50 mg/mℓ) α-casein stock solution and stored at -20 °C. The α-casein stock solution (50X) was prepared by suspending 250 mg α-casein in 5 mℓ buffer L6, aliquoted into 0.5 mℓ volumes and stored at -20°C.

### Washing buffer

Washing buffer was made up by dissolving 120 g GuSCN and 100 mℓ of 0.1 M Tris-HCl (pH 6.4) in a 500 mℓ Schott bottle, heated to 60°C to dissolve the GuSCN and dispensed into 50 mℓ Eppendorf tubes. Thereafter, 0.5 mℓ celite suspension (Section 2.1) was added to each tube to remove contaminating DNA from the buffer as described above.

### Washing ethanol

A 70% (v/v) ethanol solution was prepared with sterile distilled water.

### Preparation of spin columns [2]

The cap off 0.5 mℓ Eppendorf tubes were cut leaving the small tail behind. Several holes were made in the bottom of the tube with a red-hot needle. Important to note the holes should not be too small or too big, otherwise the filters will get blocked or the celite solution will run out of the holes and not be retained. Silica membranes were cut from GF/F filter paper using 5 mm punch. Two membranes were tightly inserted into an Eppendorf tube. The tubes are sterilized in glass jars for 15 min at 121°C.

## Modification to the In-house DNA extraction method

The DNA extraction method used as starting point for the experiments was based on the method reported by Omar et al [17]. who used a modification of the Boom et al [1]. and Borodina et al [2]. protocols. The modified in-house DNA extraction method used a combination of guanidium thiocyanate and a silicon dioxide-based material (such as celite) to lyse bacterial cells and bind genomic and plasmid DNA [1] in homemade spin columns prepared as reported by Borodina et al [2]. The spin columns added a second DNA binding step to increase the DNA yield.

Briefly the methodology is as follows:

An overnight bacterial culture (1 mℓ) was pelleted by centrifugation at 13,000 rpm for 2 min. To this pellet, 700 μℓ of lysis buffer L6 was added and incubated at 70°C for 10 min. Then 50 μℓ of celite was added to the mixture. After a 10 min incubation at room temperature (with occasional mixing), 100% ethanol was added and incubated at 70°C for 10 min. The suspension was transferred into the prepared spin columns. The spin columns were centrifuged at 13,000 rpm for 30 s. The celite pellet was subsequently washed twice with 1 mℓ of washing buffer followed by centrifugation at 13,000 rpm for 30 s. The supernatant was discarded, and the process repeated. Two more wash steps with 70% (v/v) ethanol followed this. After removing the ethanol, the pellet was dried at 56°C for 10 min. Thereafter, 100 μℓ elution buffer was added, the pellet re-suspended and the mixture heated for 10 min at 56°C followed by a final centrifugation step for 2 min at 13,000 rpm to separate the celite and the elution buffer containing the DNA.

**Fig. 1:**
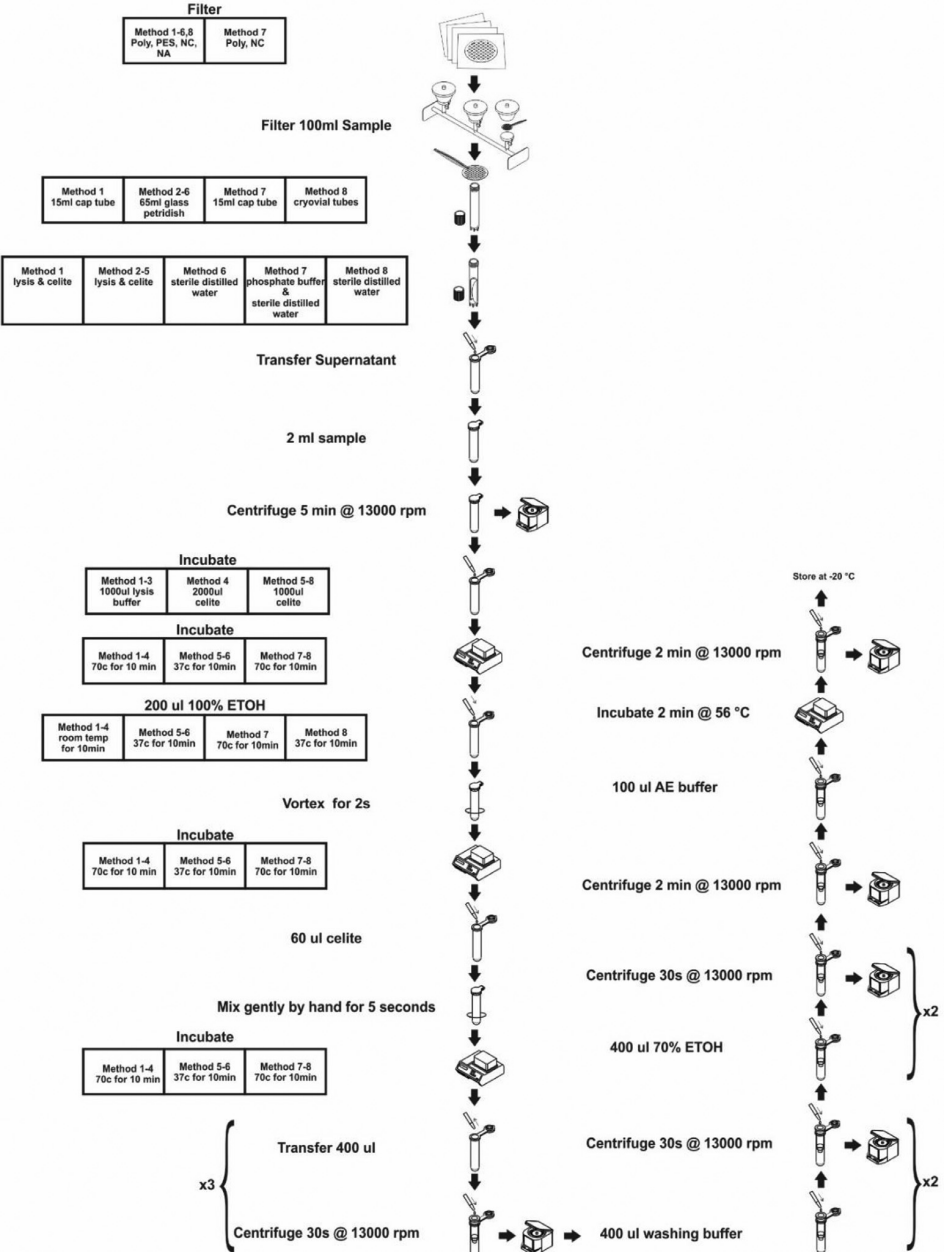
Summary of the changes made to the existing in-house DNA extraction method.

A summary of the changes made, and arguments that resulted in these changes can be seen in Table 1 and Fig. 1 but will be described below as well.

**Table 1:** Summary of the thought process, changes made, and results obtained with the in-house DNA extraction methods.

Methods	Consumables	Reason for changes in method	Summary of results
Method 1 (Section 3.1)	Filtration: Phosphate bufferMembranes: PES, Poly, NC, NA^1^Containers: 15 mℓ Falcon tube (Lasec, Cat. Log. No. 188261)	The cons of this method were: 1 Human handling of the membrane, which increases the risk of contamination when placing the membranes into the 15 mℓ Falcon tubes as well as when removing the membrane. 2 Difficulty in removing the membranes from the tube especially for Poly and NA membrane, therefore losing some of the celite.	1 Percentage DNA yield for Poly (28%) and NC (27%) membranes was ∼quarter of the reference method. 2 Comparing only the membranes Poly (85%), NC (83%), PES (55%) and NA (45%) membranes. 3 Poly and NC showed variability while NA and PES with low DNA yields were repeatable.
Method 2 (Section 3.2)	Filtration: Phosphate bufferMembranes: PES, Poly, NC, NA^1^Containers: 65 mℓ plastic petri-plates (Lasec, Cat. Log. No. GSIM2290612SA)	The pros of this method were: 1 No human handling of the membrane thus, reducing the risk of contamination. 2 The membrane does not need to be removed from the petri-dish. However, further optimization was required in reducing the evaporation of the lysis buffer in the first three steps so that more lysis buffer does not need to be added. Further optimization was also necessary to retrieve as much DNA back as compared to the PTC.	1 Percentage DNA yield increased for Poly (48%) but was still below 50% to the reference method. 2 Comparing only the membranes Poly (72%), NC (5%), PES (30%) and NA (8%) membranes. 3 By changing containers from 15 mℓ Falcon tube to 65 mℓ plastic petri-plates a reduction of DNA yield for NC and NA, PES remained the same and Poly DNA yield increased.
Method 3 (Section 3.3)	Filtration: Phosphate bufferMembranes: PES, Poly, NC, NA^1^Containers: 65 mℓ plastic petri-plates	The Poly membrane have been consistent in all three experiments. What still requires attention is in filtration since a 5^th^ of the DNA yield is lost compared to centrifugation, this has been consistent in all three experiments.	1 Reduction of DNA yield for PES (12%) membranes and an increase in DNA yield for NA (20%) and NC (90%) and Poly (75%) membranes. 2 DNA yield was low for NA but was repeatable when compared to NC, PES and Poly.
Methods	Consumables	Reason for changes in method	Summary of results
Method 4 (Section 3.4)	Filtration: Phosphate bufferMembranes: PES, Poly, NC, NA^1^Containers: 65 mℓ glass petri-plates	In this method the blank provided positive results and the DNA yield recovered compared to the positive control was the lowest.	1 With the changes made to this method, percentage DNA yield for Poly was only 5% when compared to the reference method. 2 Comparing only the membranes Poly (80%), NC (48%), PES (12%) and NA (20%) membranes.
Method 5 (Section 3.5)	Filtration: Phosphate bufferMembranes: PES, Poly, NC, NA^1^Containers: 65 mℓ glass petri-plates	This method minimized handling of the membranes and carry over contamination. However, the DNA yield was still low compared to the PTC.	1 Percentage DNA yield increased for Poly (60%) when compared to the reference method. 2 Comparing only the membranes Poly (75%), NC (2%), PES (2%) and NA (1%) membranes. 3 Even though DNA yield increased for Poly membrane, fluctuations in repeatability was a concern.
Method 6 (Section 3.6)	Filtration: Phosphate bufferMembranes: PES, Poly, NC, NA^1^Containers: 65 mℓ glass petri-plates	This method minimized handling of the membranes and carry over contamination. For PTC3, this control was discarded at step 10 because the suspension was too slimy and was blocking the membranes.	1 Percentage DNA yield increased for Poly (70%) when compared to the reference method. 2 Comparing only the membranes Poly (75%), NC (60%), PES (1%) and NA (62%) membranes. 3 Washing the membrane with sterile distilled water increased DNA yield for Poly, NC and NA but decreased for PES membrane
Method 7 (Section 3.7)	Filtration: Phosphate bufferMembranes: Poly, NC^1^Containers: 15 mℓ Falcon tubesWash membranes: Phosphate buffer and sterile distilled water	The reason for not choosing NA and PES filters is because in all 6 experiments they provided the lowest yields, further for this experiment NA would have disintegrated when vortexing making the suspension milky which would have blocked the filter tubes. It was experimented with washing the filters with either distilled water or phosphate buffer.	1 Percentage DNA yield increased for Poly (100%) when compared to the reference method. 2 Comparing only the membranes Poly (100%) and NC (44%) membranes.
Methods	Consumables	Reason for changes in method	Summary of results
Method 8 (Section 3.8)	Filtration: Phosphate bufferMembranes: Poly,NA, PES, NC^1^Containers: 4 mℓ Ogreiner Bio-1 cryovial(Lasec, Catalogue no. 1272610)Wash membranes: sterile distilled water	This method provided good recovery of DNA yield, also minimized handling of the membranes and carry over contamination. This optimised method together with Poly^1^ membrane used in further experiments since Poly membrane provided constant results compared to the remaining three membranes.	1 Percentage DNA yield dropped for Poly (80%) when compared to the reference method. 2 Comparing only the membranes Poly membranes provided same DNA yield as in method 7. DNA yield for NA membrane increased while DNA yield for NC and PES membrane decreased. 3 Repeatability for Poly and NA fluctuated and only NC with low DNA yield was repeatable.

1 PES – Polyether-sulphone, NA – Nitrocellulose-acetate, Poly – Polycarbonate, NC – Nitrocellulose ^2^PTC – Positive control

### Method 1

The major changes to the Omar et al [17]. method are described below.

Approximately 20 mℓ of phosphate buffer was filtered onto four types of membranes, namely Polycarbonate- (Poly), Nitrocellulose- (NC), Nitrocellulose-acetate- (NA) and Polyether-sulphone- (PES) membranes. This was used as the blank or negative DNA extraction control (NTC). The positive extraction control (PTC) consisted of 1 mℓ of overnight ComEC culture centrifuged at 13,000 rpm for 2 min with the DNA extracted as stated in the DNA extraction method being tested. These controls were included in the subsequent experiments below. Controls and sample analyses were performed in triplicate.

Twenty millilitre phosphate buffer, together with 1 mℓ overnight ComEC culture, was filtered onto Poly, NC, NA and PES membranes. The spin columns were placed with sterile forceps into 15 mℓ sterile Eppendorf tubes, to which 1000 uℓ lysis buffer L7A was added. The same was done for the tubes containing the positive control pellet and the negative control tube. To this 60 uℓ of the celite solution was added using a cut tip. The samples were vortexed and incubated at 70°C for 10 min with 200 rpm rotation. Following incubation, 200 uℓ of 100% ethanol was added to each tube, vortexed and incubated at room temperature for 10 min. After incubation, the PES and NC membranes were removed and discarded with pipette tips and the Poly and NA membranes were squashed on the side of the tube. This was done since it is easier to press the Poly membrane against the tube wall, and the NA membrane disintegrates in contact with lysis buffer. Approximately 400 uℓ of the sample was transferred with cut tips into the prepared spin columns and centrifuged at 13,000 rpm for 1 min to pellet the celite. The collection tubes were emptied into designated DNA waste containers and the above step repeated twice. This was followed by two more wash steps using 400 uℓ of wash buffer followed by centrifugation at 13,000 rpm for 30 s. The collection tubes were emptied into the designated DNA waste container and the above step repeated. Thereafter, 400 uℓ of 70% (v/v) ethanol solution was added to each spin column and centrifuged at 13,000 rpm for 30 s. The collection tubes were emptied into designated DNA waste container and the above step repeated. After discarding the supernatant of the second repeat, the pellet was dried by centrifuging the tubes at 13,000 rpm for 3 min. The spin columns were placed into new labelled 1.5 mℓ Eppendorf tubes and 100 uℓ of elution buffer was added to each spin column and incubated at 37°C for 2 min. After incubation, the samples were centrifuged at 13,000 rpm for 2 min during which the DNA was eluted into the Eppendorf tubes.

The DNA was quantified using Nanodrop technology (ND-1000, Inqaba Biotechnologies®) as specified by the manufacturer. The number of bacterial cells that filtered on the filters and the amount of DNA that yielded at the end of the DNA extraction method was compared to a positive extraction control (PTC).

### Method 2

The same DNA extraction protocol described in Section 3.1 was used, with the exceptions that the membranes were placed in 65 mℓ plastic petri-dishes with sterile forceps with the filtered side facing upwards instead of 15 mℓ Eppendorf tubes (Fig. 1). Lysis buffer L7A was added on top of the membrane in the plastic petri-dishes followed by the addition of 60 uℓ of the celite solution. The samples were placed in a shaking incubator at 70°C for 10 min with 150 rpm rotation. Thereafter, 200 uℓ 100% ethanol was added to each membrane. Samples were mixed with gentle swirling and incubated at room temperature for 10 min. This suspension was transferred into the spin columns and the protocol followed as described in Section 3.1.

### Method 3

The DNA extraction protocol described in Section 3.2 (Method 2) was followed with the exception that 1000 uℓ lysis buffer L7A and 60 uℓ celite was mixed, centrifuged for 1 min at 2000 rpm to pellet the celite and the supernatant used as Lysis buffer L7A. Lysis buffer L7A (2 mℓ) was removed from the membrane and placed into appropriately labelled 2 mℓ Eppendorf tubes. To this 60 uℓ of the celite solution, was added mixed and incubated at 70°C for 10 min. The remaining protocol followed as described above in Section 3.2 (Method 2).

### Method 4

The DNA extraction protocol described in Section 3.3 (Method 3) was followed, with the exception that 65 mℓ glass petri-dishes was used instead of plastic petri-plates (Fig. 1). Lysis buffer L7A (2 mℓ) was added to the positive control pellet, negative control tube and on top of the membrane in the glass petri-dishes. This was incubated in a shaking incubator at 70°C for 10 min at 90 rpm. Thereafter, 2 mℓ of the lysis buffer was removed from the membrane and placed into appropriately labelled 2 mℓ Eppendorf tubes. To this 60 uℓ of the celite, solution was added, mixed and incubated at 70°C for 10 min. The remaining protocol was followed as described above in Section 3.2 (Method 2).

### Method 5

The DNA extraction protocol described in Section 3.4 (Method 4) was followed, with the exception that Lysis buffer L7A, celite and 100% ethanol was incubated in the shaking incubator at 37°C for 10 min at 90 rpm. The remaining protocol was followed as described in Section 3.2 (Method 2).

### Method 6

The DNA extraction protocol described in Section 3.5 (Method 5) was followed, with the exception that 2 mℓ of sterile distilled water was added to each membrane and allowed to stand for 5 min. Thereafter the cells were washed from the membranes and transferred into appropriately labelled 2 mℓ Eppendorf tubes. The tubes where centrifuged at 13,000 rpm for 2 min and the supernatant was discarded. Lysis buffer L7A (1000 uℓ) was added to the pellets and negative control tube. The remaining protocol was followed as described in Section 3.2 (Method 2).

### Method 7

The DNA extraction protocol described in Section 3.6 (Method 6) was followed, with the exception that the filters were placed with sterile forceps into 15 mℓ Eppendorf tubes. The filters were vortexed for 2 min with either 2 mℓ sterile phosphate buffer or with 2 mℓ sterile distilled water. The suspension was transferred into 2 mℓ Eppendorf tubes. The tubes where centrifuged at 13,000 rpm for 10 min and the supernatant discarded. Lysis buffer L7A was added to the pellets and negative control tube. Lysis buffer, celite suspension and 100% ethanol was incubated at 70°C for 10 min. The remaining protocol was followed as described in Section 3.2 (Method 2).

### Method 8

The DNA extraction protocol described in Section 3.7 (Method 7) was followed, with the exception that the filters were placed in 4 mℓ Ogreiner Bio-1 cryovials instead of 15 mℓ Eppendorf tubes. The overnight ComEC culture (1 mℓ with ∼10–20 mℓ distilled water) were filtered onto Poly, NC, NA and PES membranes in triplicate. The filters were placed into 4 mℓ Ogreiner Bio-1 cryovial with sterile forceps. The filters were vortexed for 2 min with 2.5 mℓ sterile distilled water. The suspension was transferred into 2 mℓ Eppendorf tubes, centrifuged at 13,000 rpm for 10 min and the supernatant discarded. Lysis buffer L6 (1000 uℓ) was added to the pellets and negative control tube. The remaining protocol was followed as described in Section 3.2 (Method 2).

## ‘Method validation’ of the optimized DNA extraction method

This was an exploratory study to see if PCR can complement Microbiology, but it was decided to investigate if the method could meet some of the South African Bureau of Standards (SABS) and South African National Standard [SANS 241:2015] validation requirements. This required that the time sensitivity or robustness, precision, trueness and relative recovery, detection limit, selectivity of a method, linearity, repeatability and reproducibility, limit of quantification (where applicable) be tested. For this study, sensitivity or robustness, upper and lower detection limit (detection limit), specificity, recovery efficiency, repeatability and reproducibility were tested. Reproducibility for this study was defined as experiments repeated on three separate days instead of the use of different laboratories as described in SABS and SANS.

### DNA extraction from environmental samples

Dam water samples were collected from Emmarentia dam (Johannesburg), and borehole water samples were collected from Roodepoort (Johannesburg). Water samples were collected in 1 ℓ sampling bottles and kept on ice on route to the laboratory. Samples were analysed within 3 h from the time they were collected.

The experiments were designed to test the impact of high bacterial load (dam water) and low bacterial load (Borehole water) on four occasions as described in Table 2. These experiments were performed in triplicate with repeats on three separate days to test for repeatability and reproducibility to give a total number of 72 samples analysed. DNA was extracted using the optimized DNA extraction method described in Section 3.8 (method 8) using the Poly membrane for filtration. The number of bacterial cells that filtered and the amount of DNA that yielded at the end of the DNA extraction method was compared to a positive extraction control (cells) whereby the bacterial cells were centrifuged before DNA extraction.

**Table 2:** Experimental setup for validation on optimised DNA extraction method.

Experimental sample		Cells	Water		Explanation
			Vol. (mℓ)	Type	
High water pollution load	Set 1	No cells added	100	Sterile distilled water	Serves as no template control (NTC2)
	Set 2	No cells added	100	Dam water	Serves as water sample
	Set 3	1 mℓ EHEC cells added	99	Sterile distilled water	Serves as PTC for DNA extraction
	Set 4	1 mℓ EHEC cells added	99	Dam water	Serves as spike water sample
Low water pollution load	Set 1	No cells added	100	Sterile distilled water	Serves as no template control (NTC2)
	Set 2	No cells added	100	Borehole water	Serves as water sample
	Set 3	1 mℓ EHEC cells added	99	Sterile distilled water	Serves as PTC for DNA extraction
	Set 4	1 mℓ EHEC cells added	99	Borehole water	Serves as spike water sample

### Results

**Fig. 2:**
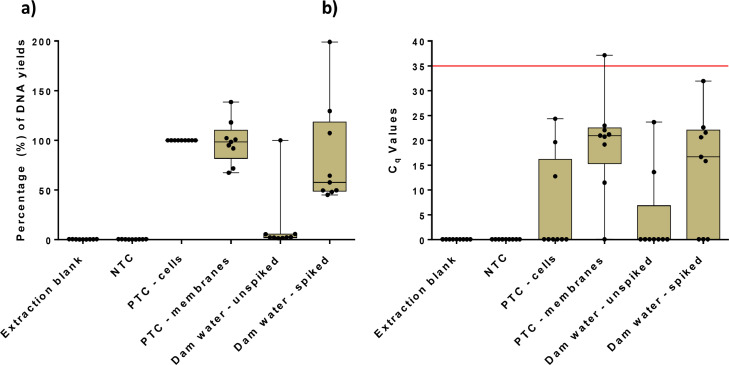
(a) Comparing percentages for DNA yields in triplicates with 3 repeats for the dam water, (b) Comparing q-PCR C_q_ values in triplicates with 3 repeats for the dam water.

**Fig. 3:**
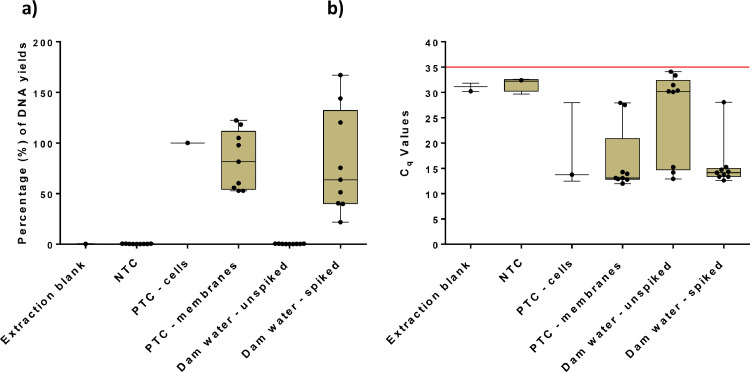
(a) Comparing percentages for DNA yields in triplicates with 3 repeats for the borehole water. (b) Comparing q-PCR C_q_ values in triplicates with 3 repeats for the borehole water.

The optimized DNA extraction method was used to test water with high (dam water) or low (borehole water) bacterial loads to determine the upper and lower detection limits of the method. When the dam water and borehole water results were compared, it highlighted the variability of the DNA extraction efficiency between 1) Extracted DNA from centrifuged bacterial cells (cells) (Figs. 2 and 3); 2) Bacterial cells were filtered on Poly membrane and then DNA was extracted on the bacterial cells that was washed from the membrane (PTC) (Figs. 2 and 3). From the statistical results, the Paired T-test, and Mann-Whitney U test (Table 3) for spiked dam water and borehole water, no-significant differences were obtained in the quantification of DNA yield and q-PCR. When compared to un-spiked dam water and borehole water, significant differences were obtained in quantification of DNA yield and no-statistical difference for q-PCR. This indicated that it was better to spike the water samples when filtering environmental water samples, which can aid in diluting inhibitors and not inhibit the q-PCR analysis.

**Table 3:** Statistical analysis for the samples with high- (dam water) and low (borehole water) bacterial loads.

Method		Paired *T*-test *P*-value	Mann-Whitney U *P*-value
Spiked water samples	DNA concentration %	0.3049**	0.2572**
Un-spiked water samples	DNA concentration %	0.0121*	0.0495*
Spiked water samples	C_q_	0.0359*	0.0495*
Un-spiked water samples	C_q_	0.3727**	0.5127**

NB: *P* ≥ 0.05 non-statistically different**; *P* ≤ 0.05 statistically different*

Furthermore, the in-house DNA extraction method was compared with commercially available water testing kits, this paper to be submitted for publication.

## Declaration of Competing Interest

The authors declare that they have no known competing financial interests or personal relationships that could have appeared to influence the work reported in this paper.

## Background

Currently routine water analysis laboratories use traditional culture base methods, which are based on chromogenic and fluorogenic media, to enumerate Commensal *E. coli* (ComEC). In the context of this study, these media have limitations, these are: (a) Low specificity is a problem, as interference from other microorganisms can occur, resulting in false-positive outcomes [19,21]. (b) Furthermore, it is un-certain if these types of media can identify DEC. DEC consists mainly of the E. coli pathotypes Entero-pathogenic *E. coli* (EPEC), Entero-toxigenic *E. coli* (ETEC), Entero-aggregative *E. coli* (EAEC), Entero-haemorrhagic *E. coli* (EHEC) and Entero-invasive *E. coli* (EIEC), Diffusely adherent *E. coli* (DAEC), Entero-aggregative-haemorrhagic *E. coli* (AEHEC) [5,8,11]. To get at these organism types, further conformational steps are required after culturing such as biochemical tests, immunological assays and polymerase chain reaction. This leads to increased cost and time. Another important consideration is that culture-based methods are unable to detect *E. coli* (both ComEC and DEC) in what is termed the viable but non-culturable (VNBC) state [13,18,19]. These are bacterial cells that are metabolically active but cannot be cultured using normal microbiological techniques. These cells are reported to be able to resume active growth when favourable conditions are restored [4,10]. The implication of this is an underestimation of total *E. coli* (TEC) numbers enumerated from a sample. Finally, it is important to detect potential DEC in the VBNC state in water because VBNC pathogenic bacteria retain their virulence genes thus making a water source a potential reservoir of disease [6,7,12]. This study was therefore, aimed to develop culture independent PCR based methods to analyse water samples for detection, characterization, and quantification of *E. coli* bacteria. Because of the cells entering VBNC state causing traditional culture methods to underestimate the pollution load, additional techniques are required to detect ComEC as well as DEC in water [12,13]. This can be achieved by techniques usually developed in the domain of molecular biology. Molecular biology techniques have been used to detect DEC types with the polymerase chain reaction (PCR) assay that targets specific genes associated with the bacterium's virulence [9,22]. PCR is frequently used because this method gives rapid and reliable results with a high sensitivity and high specificity [14,16].

Successful and routine application of PCR diagnostics is often limited by the lack of yield and quality template due to inefficient DNA extraction methodologies. Therefore, it is important to consider the following factors such as Viable but non-culturable cells; direct versus indirect DNA extraction; inhibitors; genomic DNA versus plasmid DNA. Substantial time requirements often render traditional bacterial DNA extraction (such as phenol-chloroform) impractical as additional “clean-up” procedures may be required to remove carry-over phenols which inhibit the PCR reaction. Other methods have been developed that usually involves the lyses of bacteria and subsequent binding of released DNA onto a solid matrix, followed by washing and elution of the relevant components [20]. An example of these methods is the Guanidium thiocyanate (GuSCN) method [1,15]. This method utilises chemical lyses for breaking open bacterial cells. Chemical lysis generally involves one agent attacking the cell wall and another disrupting the cell membrane. Weakening of the cell wall of *E. coli* and related organisms are usually brought about by the lysozyme enzyme; ethylenediamine tetraacetate (EDTA), or a combination of both. For this study EDTA was used on its own to remove magnesium ions that are essential for preserving the overall structure of the cell envelope and inhibits cellular enzymes that could degrade DNA [1]. Under some conditions, it is not sufficient to only use EDTA to weaken the cell wall. Therefore, a detergent such as Triton X-100 were added. Detergents aid the process of lysis by removing lipid molecules and thereby causing disruption of the cell membranes [3]. Another important consideration is the biochemical content of the cells from which DNA is being extracted. With most bacteria the main biochemicals present in a cell are protein, DNA and RNA. Guanidium thiocyanate is a compound which has two properties that makes it useful for DNA purification. First, it denatures and dissolves all biochemicals other than the nucleic acids and can therefore be used to release DNA from virtually any type of tissue. Secondly, in the presence of this compound, DNA binds tightly to silica or celite particles. This provides an easy way of recovering the DNA from the denatured mix of biochemicals [1]. What is also very important is that the DNA binding to the silica or celite particles is influenced by salt concentration – the more salt the greater the binding potential. This is a major part of the silica method as the nucleic acid is bound to the silica and washed using solutions with a high salt concentration and then eluted using a solution with a low salt concentration such as TE buffer or distilled water [3]. GuSCN has shown to be a powerful agent in the purification and detection of both RNA and DNA because of its potential to lyse cells combined with its potential to inactivate nucleases [3].

The decision was made for this study to use the GuSCN method by Boom et al [1]. and Borodina et al [2]. (since majority of non-commercial DNA extractions and commercial kits are based on these methods) and modify this method for extracting DNA directly on the filters that have concentrated bacteria from water.
